# Dr. Storer on Dr. Simpson

**Published:** 1870-06

**Authors:** 


Dr. Storer on Dr. Simpson.—Dr. R. H. Storer, of Boston, at a meeting of Medical men at the Army Medical Museum, Washington, made the following remarks pertinent to the intelligence of the death of his friend, Dr. Simpson, of Edinburgh:

" *Mr. Chairman and Brethren of the Profession:* It is with the deepest grief that I announce to you the decease, of which I have learned by cable dispatch from his son, Professor Sir James Y. Simpson, of Edinburgh. That our friend had himself prepared me by messages from his dying bed for his departure renders the loss none the easier to be borne. His very fortitude in his suffering, and his resignation to the Divine will, but revealed a more profound depth and a lovelier beauty in his character, with which it was but the harder to part.

" Dr. Simpson was so universally known to our profession throughout the world, as he was, indeed, to mankind at large, that I need enter into no detail concerning his history. He rose from the obscurity of a country village to the favorite of his Sovereign, the peer of the highest literary and scientific authorities, the cynosure of the medical and surgical intellect of this century. It was as if by magic, but his only talisman lay in the perfect bravery, persistency, sincerity and simplicity of his life. Quick to perceive, he was equally apt in executing. He expended no unnecessary force, he begrudged no required effort. He was not merely the skilled accoucheur and the thoughtful, wise gynæcologist. His suggestions regarding acupressure, now in daily practice, have placed him as a surgeon side by side with Ambrose Pare, while the revolution achieved by him as to the fundamental idea of hospitalism, entitles him to the glorious appellation of having been the modern father of medicine, a second Hippocrates.

" There are those present who, from personal acquaintance with the facts, know that these claims are in no sense forced or over stated. At less than sixty he had accomplished all that has been said; and yet, still in the fresh prime of his life, we hoped for even riper fruits from his vast experience. But it was not so ordained. His last professional work, sent to us from his death-bed, was for the vindication of the honor of one of our own countrymen, whose memory, thus redeemed, will always be embalmed in our hearts, conjoined with that of Simpson. His reply to the second letter of Dr. Jacob Bigelow concerning the history of practical anæsthesia, communicated to the Gynæcological Society, of Boston, and received through it by you at the session of the American Medical Association just ended, went far to influence your decision as to the person to whom the honor of that glorious discovery, the most beneficent ever made since the foundation of the world, was really due. By a unanimous vote upon the last day of the session, and in pursuance of the recommendation of the Section of the Practical Medicine, by whom the evidence adduced had been carefully scrutinized, you pronounced in favor of the late Dr. Horace Wells, of Hartford, Connecticut. Upon the evening of the same day—his earthly labors thus beautifully ended—the spirit of Dr. Simpson took its flight.

"There would be no place in this country so fitting for these few words of eulogy, this poor utterance of our common gratitude, as the national capital; and this Army Medical Museum, filled as it is with the results of achievements formerly impossible, is in itself his fitting monument. Coming together from the uttermost parts of the continent, we go hence to homes where his name is everywhere a household word, never to be forgotten so long as the primal curse, from which, through God's great grace, he took the sting, shall lie upon suffering women. The nations shall rise up, indeed, to call him blessed, and blessed he is, if to have faithfully worked through his whole life in the Lord's vineyard, in season and out of season, cheerfully doing that which was given him to do, bearing up under the heaviest domestic afflictions, not resenting unkindly the sarcasms and ingratitude of petty men; assuming withal, for the dear Savior's sake, his heavy cross, the jeers of sceptics and the taunts of those who daily crucify Him anew—if to have lived thus is to have gained entrance into the joy of the Lord, blessed is he indeed.

"Of my own personal bereavement I have now nought to speak. There may be those present, however, who will remember my language of fifteen years ago, in the preface to the American edition of those 'Memoirs and Contributions,' to edit which, in conjunction with Dr. Priestley, now of London, it had been my great privilege to be selected. 'Treating me as his son, I had learned to love him as a parent.' As such, indeed, I have found the tie that is now broken.."

